# Homocysteine Inhibits Hepatocyte Proliferation via Endoplasmic Reticulum Stress

**DOI:** 10.1371/journal.pone.0054265

**Published:** 2013-01-22

**Authors:** Xue Yu, Jiajun Lv, Yunzhen Zhu, Liping Duan, Lanqing Ma

**Affiliations:** 1 Department of Cardiology, Beijing Hospital, Ministry of Health, Beijing, China; 2 Department of Digestive Diseases, The First Affiliated Hospital, Kunming Medical University, Kunming, Yunnan, China; St.Louis University, United States of America

## Abstract

Homocysteine is an independent risk factor for coronary, cerebral, and peripheral vascular diseases. Recent studies have shown that levels of homocysteine are elevated in patients with impaired hepatic function, but the precise role of homocysteine in the development of hepatic dysfunction is unclear. In this study, we examined the effect of homocysteine on hepatocyte proliferation in vitro. Our results demonstrated that homocysteine inhibited hepatocyte proliferation by up-regulating protein levels of p53 as well as mRNA and protein levels of p21^Cip1^ in primary cultured hepatocytes. Homocysteine induced cell growth arrest in p53-positive hepatocarcinoma cell line HepG2, but not in p53-null hepatocarcinoma cell line Hep3B. A p53 inhibitor pifithrin-α inhibited the expression of p21^Cip1^ and attenuated homocysteine-induced cell growth arrest. Homocysteine induced TRB3 expression via endoplasmic reticulum stress pathway, resulting in Akt dephosphorylation. Knock-down of endogenous TRB3 significantly suppressed the inhibitory effect of homocysteine on cell proliferation and the phosphorylation of Akt. LiCl reversed homocysteine-mediated cell growth arrest by inhibiting TRB3-mediated Akt dephosphorylation. These results demonstrate that both TRB3 and p21^Cip1^ are critical molecules in the homocysteine signaling cascade and provide a mechanistic explanation for impairment of liver regeneration in hyperhomocysteinemia.

## Introduction

Homocysteine is an intermediate in sulfur amino acid metabolism. Elevated levels of circulating homocysteine, namely hyperhomocysteinemia, has been regarded as an independent risk factor for atherosclerosis [1]–[3]. Homocysteine can induce cell cycle arrest [4], senescence [5], and apoptosis in endothelial cells [6] and neurons [7].

One of mechanisms of homocysteine-induced cellular dysfunction involves endoplasmic reticulum (ER) stress [3], [4], [6]. Homocysteine may cause ER stress by disrupting disulfide bond formation and activating the unfolded protein response. When human umbilical vein endothelial cells are exposed to supraphysiological concentrations of homocysteine (1–5 mM), the expressions of ER stress response genes, such as GRP78/BiP(78-kDa glucose-regulated protein), CHOP/GADD153 (CEBP homology protein/growth arrest and DNA damage-inducible protein 153), and ATF4 are up-regulated [4]. Although the precise mechanism by which ER stress promotes cell cycle arrest and/or apoptosis is not fully understood, increased CHOP expression has been implicated in the commitment to cellular dysfunction [4], [8], [9]. The overexpression of CHOP enhances ER stress-induced cell growth arrest and/or apoptosis [10], [11]. In contrast, CHOP-deficient mice exhibit reduced apoptosis in response to ER stress [10].

Liver is a major organ in homocysteine metabolism. Recently, epidemiological and experimental studies also link hyperhomocysteinemia to a wide range of impaired liver function. For instance, elevation of homocysteine has been observed in patients with cirrhosis and chronic alcohol consumption [12], [13]. Additionally, elevated homocysteine levels also appear in experimental anmial models of ethanol and CCl4-induced hepatotoxicity [14], [15]. On the other hand, patients with methylenetetrahydrofolate reductase (MTHFR) C677T polymorphism have high levels of homocysteine in plamsa, leading to hepatic steatosis and fibrosis [16]. It has been shown that hyperhomocysteinemia can alter intracellular lipid metabolism [17]. Homocysteine stimulates the biosynthesis of cholesterol via up-regulation of a transcription factor, sterol regulatory element–binding protein-1 [18]. Cystathionine β-synthase (CBS) -deficient mice with hyperhomocysteinemia develop fibrosis [19]. Moreover, our recent studies have shown that hyperhomocysteinemia disturbs hepatic glucose metabolism, resulting in insulin resistance [20], [21]. Recently, Liu et al. [22] have reported that hyperhomocysteinemia induced by methionine diet impairs liver regeneration in mice.

The fact that hyperhomocysteinemia is associated with impairment of hepatic regeneration promotes us to examine the cytotoxic effect of homocysteine on hepatocyte proliferation. Our results reveal that homocysteine induces cell cycle arrest in the G1 phase through induction of TRB3 expression, via the ER stress pathway. The p53/p21^cip1^ pathway plays a critical role in homocysteine-mediated cellular toxicity. Our data provide a molecular basis for inhibition of hepatocyte proliferation by homocysteine.

## Materials and Methods

### Cell Culture and Homocysteine Treatment

Mouse hepatocytes were prepared and characterized as described previously [23]. Hepatocytes were plated in serum-free William’s medium E (Gibco, Gaithersburg, MD), including insulin (20 mU/ml, Sigma), epidermal growth factor (50 ng/ml). The medium was changed every day thereafter. After Cells were cultured for 48 h, experiments were initiated by addition of DL-homocysteine (Sigma, St Louis, MO).

Human hepatocarcinoma cell lines HepG2 and Hep3B were obtained from the Kunming Cell Center, Chinese Academy of Sciences (Kunming, China). The cells were grown in DMEM (Gibco) with 10% fetal bovine serum (FBS) and maintained at 37°C, 5% CO_2_. When cells were 60–80% confluent, the culture medium was changed to DMEM lacking FBS and maintained for 24 h. Experiments were initiated with fresh DMEM with 5% FBS and containing DL-homocysteine (Sigma, St Louis, MO). Cells were counted in triplicate in a hemocytometer after 7 days of incubation with homocysteine. Medium and homocysteine were replaced every 2 days.

### [^3^H]-thymidine Incorporation Assay

Aliquot cell suspension was added into 24-well dishes. After incubated with homocysteine for 18 h, the cells were added 50 µl of serum-free medium containing [^3^H]-thymidine (Atom High-Tech Co., Beijing, China) at 1 µCi/ml to each well. After incubation for an additional 6 h, cells were washed with PBS three times. [^3^H]-thymidine incorporation assay was performed measured by a LKB 1214 scintillation counter (Sweden).

### Quantitative Real-time RT-PCR Analysis

Total RNA from cells was isolated using Trizole reagent (Invitrogen, Carlsbad, CA). Random-primed cDNAs were generated by reverse transcription of total RNA samples with SuperScript II (Invitrogen). A realtime-PCR analysis was performed with the ABI Prism 7000 Sequence Detection System (Applied Biosystems, Foster City, CA) using SYBR® Premix-Ex Tag™ (Takara, Dalian, China). GAPDH (for human genes) or actin (for mouse genes) was used for internal control, respectively. The primers used for PCR were listed in Table S1. The primers used for PCR were listed in Table S1.

### Western Blotting

Cells were lysed on ice for 30 min in lysis buffer (containing 0.15 M NaCl, 30 mM Tris, 1 mM phenylmethanesulfonyl fluoride, 1% Triton X-100, 1 mM EDTA, 10 µg/ml leupetin, 2 µg/ml pepstatin, 2 µg/ml aprotinin and 2 mM Na_3_VO_4_). Cell lysates (20 µg) of total protein were loaded per well and separated on a 10% SDS polyacrylamide gel. Proteins were then transferred to PVDF membranes. Primary antibodies were: anti-Akt and anti-phospho (Ser^473^) Akt, anti–actin antibodies (Sigma, St Louis, MO), anti-CHOP, anti-GRP78, anti-ATF4, anti-p21^Cip1^, anti-p53, anti-p27^kip1^ antibodies (Santa Cruz Biotech, Santa Cruz, CA), and anti-TRB3 antibodies (Calbiochem La Jolla, CA). The secondary antibody was a peroxidase-coupled anti-rabbit IgG (Amersham Biosciences, Piscataway, NJ). The membrane was exposed to ECL Hyperfilm (Amersham Biosciences), and the film was developed. Each blot was stripped with a stripping solution (0.1 M glycine, pH2.9) for 1 h and re-probed with anti-Akt or anti-actin antibodies to the bands were quantified densitometrically.

### Plasmid Construction for the Expression of CHOP

The human CHOP gene was amplified from HepG2 DNA, using primers 5′-GGG GAT CCC AGA GAT GGC AGC TGA GTC-3′ (forward) and 5′-GTC GAC GCT CCC AAT TGT TCA TGC TT-3′ (reverse). Restriction sites for *EcoR I* and *BamH I* were incorporated into the primers. The amplified fragment, was digested with *EcoR I* and *BamH I*, and was then inserted between the respective sites in pCMV3C vector (a gift from Dr. CG Zou, University of Yunnan, China) to create the recombinant plasmid, pCMV3C-CHOP.

### Transfection

Transfection was performed using Lipofectamine 2000 Reagent in OPTI-MEM medium (Invitrogen) according to the manufacturer’s specifications. In parallel experiments, the expression vectors were replaced with empty vectors. 48 h following transfection, the medium was replaced with fresh DMEM containing 10% FBS. Stable cell lines of HepG2 that express full-length CHOP or shRNA for TRB3 gene [22], were produced by transfection of pCMV3C-CHOP plasmid or pSilencer2.1-U6-shRNA followed by positive colony selection using G418 (Sigma) at a concentration of 500 µg/ml.

### Statistical Analysis

Data from experiments were expressed as mean ± SD. Statistical difference between the groups was analyzed using one-way ANOVA, followed by post-hoc comparisons using two-tailed *t-*test. Values of *P*<0.05 were considered statistically significant.

## Results

### Homocysteine Inhibits Proliferation in Hepatocytes

**Figure 1:**
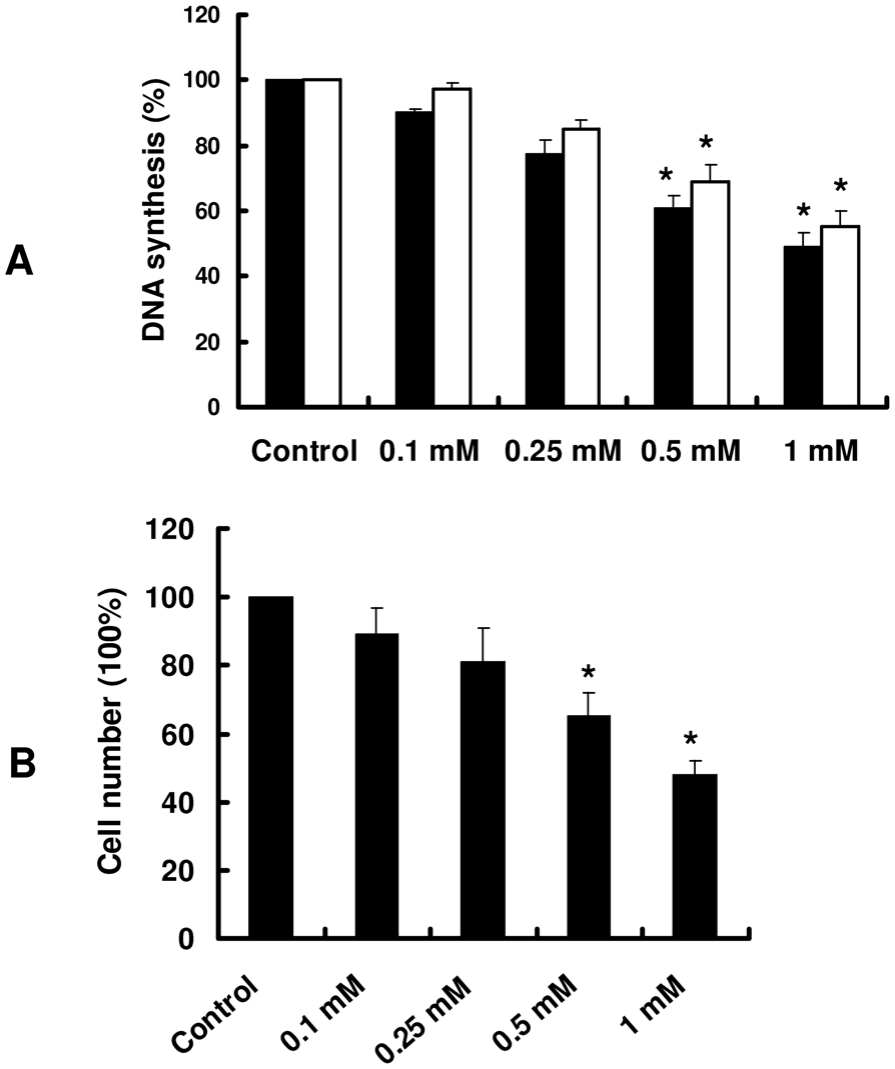
Homocysteine inhibits cell proliferation. Primary cultured hepatocytes or HepG2 cells were incubated with varying concentrations of homocysteine (Hcy). (A) The cell proliferation was assessed by [^3^H]-thymidine incorporation into DNA after treatment with Hcy for 24 h. (B) HepG2 cells were counted in triplicate in a hemocytometer after 7 days of incubation with Hcy. Medium and Hcy were replaced every 2 days. These results are means ± SD of five experiments. *P<0.05 versus control (without Hcy).

In this study, we found that homocysteine (0.1–2 mM) did not cause a significant lactate dehydrogenase leakage (an index of cell injury) for the 24 h treatment in both cultured primary hepatocytes and HepG2 cells (data not shown). The effect of homocysteine on cell proliferation was assessed by [^3^H]-thymidine incorporation into DNA. As shown in Fig. 1A, treatment of homocysteine markedly inhibited this incorporation in a dose-dependent manner in primary cultured hepatocytes and HepG2 cells. Concentrations of 0.1, 0.25, 0.5, and 1 mM homocysteine resulted in 10%, 23%, 39% and 51% decrease in [^3^H]-thymidine incorporation in hepatocytes, respectively. Meanwhile, homocysteine suppressed the proliferation of HepG2 cells as also indicated by cell numbers. By day 7, homocysteine at 0.1, 0.25, 0.5, and 1 mM reduced cell number by 11%,19%, 35% and 52%, respectively (Fig. 1B). We found that cysteine (0.1–5 mM), another thiol-containing amino acid, did not significantly inhibit hepatic proliferation, indicating this effect was selective for homocysteine (data not shown).

### p53/p21^Cip1/WAF1^ is Essential for Homocysteine-induced Cell Growth Arrest

**Figure 2:**
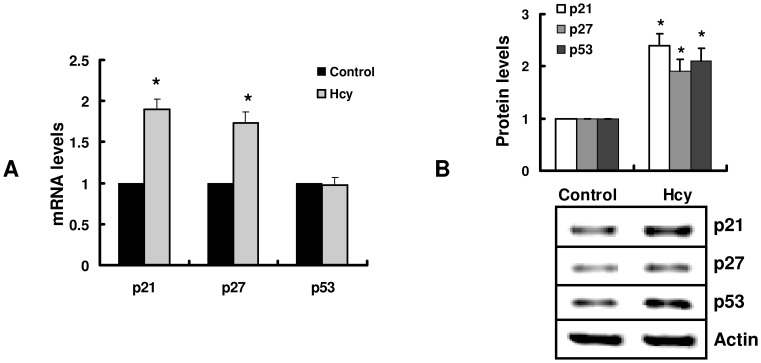
Homocysteine upregulates the expression of genes involved in transition of cell cycle. (A) Primary cultured hepatocytes were incubated with 1 mM of homocysteine (Hcy) for 8 h. The mRNA levels were detected by real-time PCR. All results are standardized to the levels of actin and are the means ± SD of five experiments. *P<0.05 versus control (without Hcy). (B) The proteins from hepatocytes were detected by Western blotting. The blot is representative of three independent experiments. The upper part shows quantification of immunoreactivity levels. The data are expressed as percent change from control. *P<0.05 versus control (without Hcy).

Since homocysteine induced cell cycle arrest, we examined the effect of homocysteine on the expression of genes involved in cell cycle. Candidate genes chosen for analysis were the Cdk inhibitor proteins p21^Cip1/WAF1^, p27^kip1^ as well as cyclins E and D1 [24]. As shown in Fig. 2A and B, the mRNA and protein levels of p21^Cip1^, p27^kip1^, were up-regulated by homocysteine in hepatocytes. In contrast, homocysteine treatment did not affect the expression of cyclins E and D1 (data not shown). Since p21^Cip1^ is a direct transcriptional target of p53, we thus examined the effect of homocysteine on the expression of p53. Homocysteine treatment resulted in an increase in the protein levels of p53, but did not alter the amount of p53 transcript in hepatocytes (Fig. 2B).

**Figure 3:**
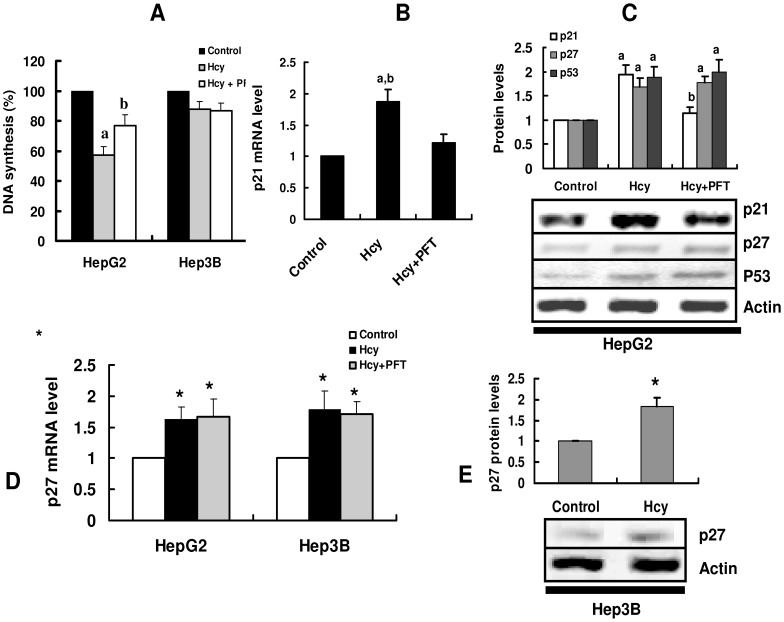
p53 and p21^Cip1^ are involved in homocysteine-mediated cell growth arrest. (A) HepG2 or Hep3B were incubated with 1 mM homocysteine (Hcy) for 24 h in the presence or absence of a p53 inhibitor, pifithrin-α (PFT) (30 μM). The cell proliferation was assessed by [^3^H]-thymidine incorporation into DNA. ^a^ *P*<0.05 versus control (without Hcy); ^b^ *P*<0.05 versus Hcy. (B) and (D) HepG2 or Hep3B were incubated with 1 mM Hcy for 8 h. The expression of genes was determined by real-time PCR. All results are standardized to the levels of GAPDH and are the means ± SD of five experiments.^ a^ *P*<0.05 versus control (without Hcy); ^b^ *P*<0.05 versus Hcy+PFT; *P<0.05 versus control (without Hcy). (C) and (E) The proteins from HepG2 or Hep3B were detected by Western blotting. The blot is representative of three independent experiments. The upper part shows quantification of immunoreactivity levels. The data are expressed as percent change from control. ^a^ *P*<0.05 versus control (without Hcy); ^b^ *P*<0.05 versus Hcy; *P<0.05 versus control (without Hcy).

To elucidate the role of p53 in homocysteine-induced hepatic toxicity, we first compared the effects of homocysteine on proliferation in p53-positive HepG2 and p53-null hepatocarcinoma cell line, Hep3B. As described above, homocysteine inhibited cellular proliferation in HepG2, but not in Hep3B cells (Fig. 3A). Furthermore, homocysteine up-regulated the protein levels of p53 as well as both the mRNA and protein levels of p21^Cip1^ in HepG2 cells (Fig. 3B,C). As expected, both the mRNA and protein levels of p53 were undetectable in Hep3B cells (data not shown). Similarly, p21^Cip1^ expression was almost not detected in Hep3B cells in the presence or absence of homocysteine (data not shown). However, homocysteine induced an increase in the expression of p27^kip1^ in both HepG2 and Hep3B cells (Fig. 3C–E). Second, we found that a p53 inhibitor, pifithrin-α [25], markedly reversed homocysteine-induced cell growth arrest (Fig. 3A) and induction of p21^Cip1^ expression in HepG2 (Fig. 3B,C). In contrast, pifithrin-α did not affect the expression of p27^kip1^ (Fig. 3C,D). Finally, we knockdown the expression of p53 by siRNA in HepG2 cells (Fig. S1A). We found that genetic inactivation of p53 significantly suppressed the expression of p21^Cip1^ (Fig. S1B and C) and led to a decrease in cellular proliferation in HepG2 (Fig. S2). Taken together, these results indicated that p53/p21^Cip1^ pathway is essential in homocysteine-induced cell cycle arrest.

### Homocysteine Upregulates TRB3 through ER Stress

**Figure 4:**
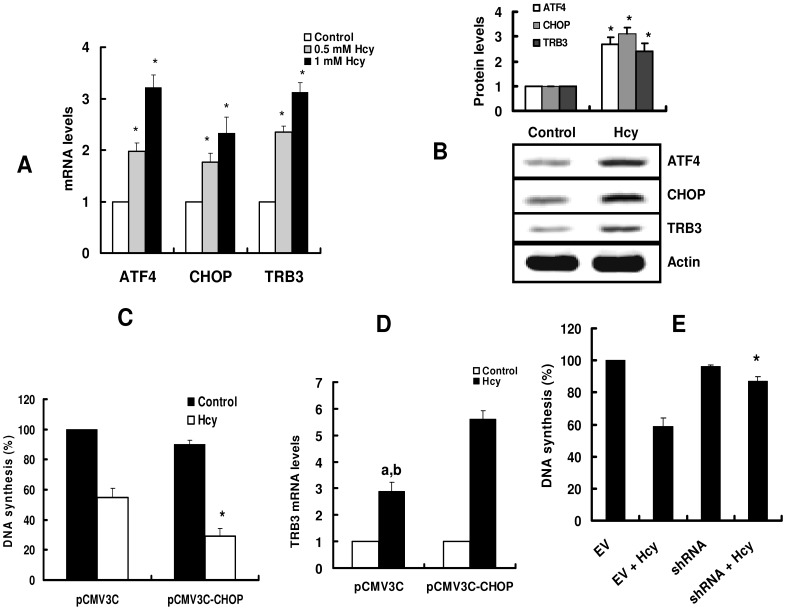
TRB3 is required for homocysteine-mediated cell growth arrest. (A) Primary cultured hepatocytes were incubated with 0.5 or 1 mM homocysteine (Hcy) for 8 h. Total RNA was extracted and subjected to real-time PCR. All results are standardized to the levels of actin and are the means ± SD of five experiments. *P<0.05 versus control (without Hcy). (B) Primary cultured hepatocytes were incubated with 1 mM Hcy for 8 h. The protein levels were detected by Western blotting. The blot is typical of three experiments. The upper part shows quantification of immunoreactivity levels. The data are expressed as percent change from control. *P<0.05 versus control (without Hcy). (C-D) HepG2 cells were transfected with pCMV3C empty vector or pCMV3C-CHOP. The cell proliferation was assessed by [^3^H]-thymidine incorporation into DNA after treatment with Hcy for 24 h (C). These results are means ± SD of five experiments. ^*^ *P*<0.05 versus pCMV3C (with Hcy). After incubated with 1 mM Hcy for 24 h, total RNA was extracted and subjected to real-time RT-PCR (D). ^a^ *P*<0.05 versus control (without Hcy); ^b^ *P*<0.05 versus Hcy +pCMV3C-CHOP. (E) Stable cell lines of HepG2 that express shRNA for TRB3 gene, were produced by transfection of pSilencer2.1-U6-shRNA. The cell proliferation was assessed by [^3^H]-thymidine incorporation into DNA after treatment with Hcy for 24 h. EV, empty vector. *P<0.05 versus EV+Hcy.

ER stress is thought to be responsible for initiating cycle arrest or apoptosis in endothelial cells by [3], [4], [6]. To clarify the molecular mechanism underlying homocysteine-induced cell growth arrest, the expression of ER stress-responsive genes was examined after homocysteine treatment. As shown in Fig. 4A and B, hepatocytes exhibited an increase in CHOP and ATF4 expression in hepatocytes. As described previously, CHOP is a critical molecule in ER-stress-induced cell growth arrest and/or apoptosis [10], [11]. To study whether enforced expression of CHOP affects cell growth arrest, we established a stable HepG2 cells overexpressing CHOP. We found that CHOP overexpression did not significantly inhibit cell proliferation by itself but sensitized these cells to a further homocysteine treatment (Fig. 4C). These data suggest that the inhibitory effect of homocysteine on cell proliferation is due to its ability to promote ER stress.

Our next step was to identify genes downstream of CHOP that could participate in the effect of homocysteine. Previous studies have identified several target genes of CHOP involved in cell survival or death. These genes includes carbonic anhydrase VI [26], death receptor 5 [27], TRB3 (tribbles-related protein 3) [28], and Bcl-XL [29]. In this study, we found that the expression of death receptor 5 and Bcl-XL remained unchanged after homocysteine treatment (data not shown). The expression of carbonic anhydrase VI was not detected (data not shown). In contrast, homocysteine significantly promoted the expression of TRB3 in hepatocytes (Fig. 4A and B). It has been reported that ATF4 and CHOP cooperate in activating the TRB3 expression [28]. We found that CHOP overexpression markedly elicited homocysteine-induced TRB3 expression in HepG2 cells (Fig. 4D).

Although homocysteine up-regulates TRB3, it is still unclear whether this induction is responsible for the cell cycle arrest. To address whether this induction of TRB3 is responsible for the inhibition of proliferation, endogenous TRB3 expression was ablated by shRNA in HepG2 cells. Proliferation was significantly inhibited by homocysteine in HepG2 cells transfected with shRNA against TRB3 mRNA (Fig. 4E). These data suggest that TRB3 is essential for homocysteine-induced cell growth arrest.

### Inhibition of Akt Activity is Responsible for Cell Cycle Arrest Induced by Homocysteine

**Figure 5:**
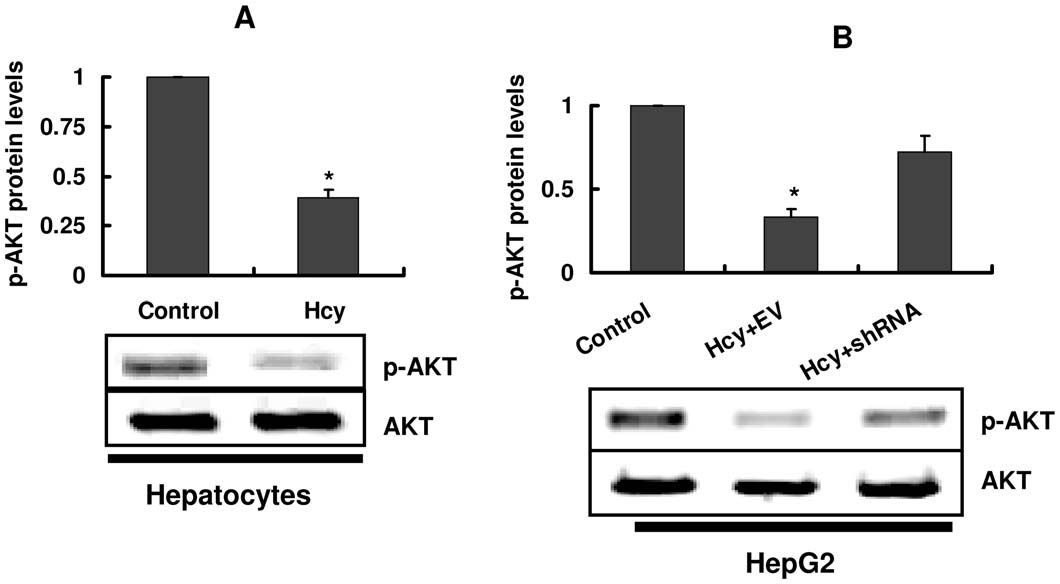
Homocysteine reduces phosphorylation of Akt. (A) Primary cultured hepatocytes were incubated with 1 mM homocysteine (Hcy) for 8 h. The protein levels were detected by Western blotting. The blot is typical of three experiments. The upper part shows quantification of immunoreactivity levels. The data are expressed as percent change from control. *P<0.05 versus control (without Hcy). (B) HepG2 cells were transfected with shRNA against TRB3 mRNA. EV, empty vector. After incubated with 1 mM Hcy for 8 h, whole cell proteins were extracted and analyzed by Western blotting with antibodies against pSer-473-Akt. The blot is typical of three experiments. The upper part shows quantification of immunoreactivity levels. The data are expressed as percent change from control. *P<0.05 versus control (without Hcy).

Akt, a serine/threonine kinase, has been found to promote cell survival and cell proliferation. Previous studies indicate that TRB3 inhibits the transmission of the Akt signal [22], [30]. Since phosphorylation of Akt at Ser473 was tightly correlated with its activation, we examined the effect of homocysteine on phosphorylation of Akt (Ser473). Homocysteine (1 mM) treatment reduced the phosphorylation of Akt (Ser473) in hepatocytes and HepG2, respectively (Fig. 5A and B). Knockdown of TRB3 by shRNA partially depressed the inhibitory effect of homocysteine in HepG2 cells (Fig. 5B). Thus, homocysteine suppressed activity of Akt by a TRB3-dependent manner.

**Figure 6:**
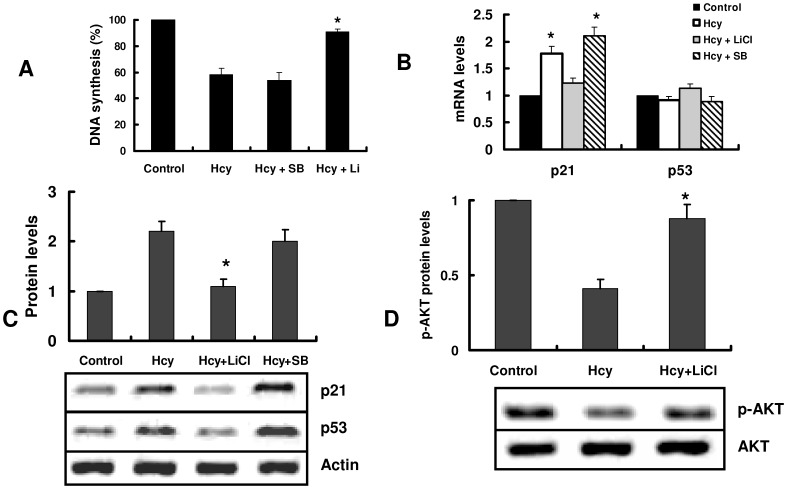
LiCl inhibits homocysteine-induced cell growth arrest through activation of Akt. Primary cultured hepatocytes were preincubated with SB216763 (10 µM) (SB) or LiCl (20 mM) before incubated with 1 mM homocysteine (Hcy). (A) After treatment with Hcy for 24 h, the cell proliferation was assessed by [^3^H]-thymidine incorporation into DNA. (B) After treatment with Hcy for 8 h, the mRNA levels were detected by real-time PCR. All results are standardized to the levels of actin and are the means ± SD of five experiments. (C) and (D) The protein levels were detected by Western blotting. The blot is representative of three independent experiments. The upper part shows quantification of immunoreactivity levels. The data are expressed as percent change from control. (C) *P<0.05 versus control (without Hcy). (D) *P<0.05 versus Hcy.

GSK3β is a major downstream target of Akt. Akt phosphorylates GSK3β to make it inactive [31]. We found that SB216763 (10 µM), a specific GSK3β inhibitor, had no effect on proliferation induced by homocysteine (Fig. 6A), indicating that GSK3β is not involved in the inhibition of cellular proliferation by homocysteine. Interestingly, a selective inhibitor of GSK3β, LiCl (20 mM), markedly suppressed the inhibitory effect of proliferation by homocysteine (Fig. 6A). To clarify the differential effects of SB216763 and LiCl, we examined the effect of these two compounds on expression of genes associated with homocysteine-mediated cell arrest. As shown in Fig. 6B and C, LiCl but not SB216763 significantly inhibited the induction of expression of p21^Cip1^ as well as p53 protein levels by homocysteine.

It has been shown that LiCl not only inhibits the activity of GSK3β, but also activates the activity of Akt through PI3K [32]. We thus examined the effect of LiCl on phosphorylation of Akt (Ser473). As shown in Fig. 6D, LiCl attenuated the inhibitory effect of homocysteine on phosphorylation of Akt (Ser473), while SB216763 had no such effect (data not shown). These results suggest that activation of Akt by LiCl inhibits homocysteine-induced cell growth arrest.

## Discussion

In this study, we demonstrate that homocysteine inhibits cell proliferation via impairment of normal cell cycle regulation in hepatocytes. Thus, our results provide important insights into the molecular mechanism underlying hyperhomocysteinemia-mediated impairment of liver regeneration.

It has been documented that homocysteine induces growth arrest or apoptosis though ER stress in endothelial cells [3], [4], [6]. Up-regulation of CHOP probably plays a potential role in linking homocysteine-mediated ER stress to alterations in endothelial growth and proliferation [4]. Likewise, elevated expression of CHOP has been considered as a critical molecule for hepatic apoptosis in a murine model of ethanol feeding [29]. However, there is still a missing link between the induction of CHOP and cellular dysfunction. In this study, we demonstrate that homocysteine (a) up-regulates TRB3 expression in a CHOP-dependent manner, (b) attenuates the phosphorylation of Akt, (c) enhances the expression of p53 and p21^Cip1^, and (d) inhibites cell proliferation. Thus, our results provide the evidence that inducible expression of TRB3 by homocysteine-induced ER stress is responsible for cell growth arrest.

TRB3 is a mammalian homologue of the Drosophila Tribbles, a protein identified as a regulator of cell cycles in Drosophila [33]. It has been shown that Tribbles mediates the degradation of string/CDC25 by the ubiquitin-dependent pathway in *Drosophil*a [33]. However, there is no evidence that TRB3 is involved in protein degradation in mammals [34]. TRB3 has been shown to interact directly with Akt and suppress the phosphorylation of this kinase in livers [30]. A recent study has confirmed that TRB3 is a novel target gene of CHOP involved in the coordination of apoptosis during ER stress [28]. Our recent study indicates that expression of TRB3 is elevated in livers of mice with hyperhomocysteinemia [22]. In the current study, homocysteine induces TRB3 expression through the ER stress pathway in cultured hepatocytes. Knockdown of TRB3 expression by shRNA partially blunts cell growth arrest in HepG2 cells. Thus, TRB3 is a critical component in homocysteine-mediated cell growth arrest in hepatocytes. He et al. [35] have reported that both the protein and the mRNA levels of TRB3 are elevated in hepatic tissues in rats fed with ethanol. The mechanisms underlying ethanol effects on TRB3 expression remain unclear. Since ethanol also induces hepatic dysfunction through the ER stress pathway, it is likely that upregulation of TRB3 is also mediated by CHOP.

It has been well established that the PI3K/Akt signaling pathway is important to promote cell survival and proliferation [36]. Suhara et al. [37] have reported that homocysteine significantly inhibits Akt activity in endothelial cells. Activation of Akt by infection of adenoviral construct expressing the constitutively active form of Akt reversed endothelial apoptosis induced by homocysteine. Although the mechanism underlying homocysteine-mediated inactivation of Akt remains unknown, these results suggests that the Akt-signaling is a novel target for homocysteine-induced endothelial cytotoxicity in vitro. In the current study, we demonstrate that homocysteine reduces phosphorylation of Akt (Ser473) through a TRB3-dependent pathway in hepatocytes, indicating that cell proliferation inhibited by homocysteine results from inactivation of Akt. The fact that LiCl significantly suppresses cell growth arrest and dephosphorylation of Akt induced by homocysteine supports this view.

We observe that the protein levels of p53 are increased after homocysteine treatment in hepatocytes and HepG2 cells. In contrast, homocysteine has no effect on cell proliferation in Hep3B, which does not express p53 gene. Furthermore, inhibition of p53 activity by its inhibitor pifithrin-α or knockdown of p53 expression significantly suppresses homocysteine-induced cell growth arrest. The fact that Akt can phosphorylate MDM2 and enhance MDM2-mediated degradation of p53 by ubiquitination [38] may provide a ready explanation of our observation that homocysteine up-regulates protein levels, but not mRNA levels, of p53. p21^Cip1^, which is a target gene of p53, plays a role in the G1 transition by inhibiting cyclin E/Cdk2 [39]. Homocysteine markedly induces the expression of p21^Cip1^ in HepG2, but not in Hep3B, indicating that p21^Cip1^ is the downstream molecule of p53 in homocysteine-induced cell growth arrest. Consistent with our in vitro results, a significant increase in expression of p53 and p21^Cip1^ is observed in livers of mice with hyperhomocysteinemia [23]. Thus, p53/p21^Cip1^ pathway is essential in homocysteine-induced cell growth arrest. Like p21^Cip1^, p27^kip1^ is also associated with a cell-cycle arrest through its inhibitory effect on cyclin E/Cdk2 complexes [40]. Although homocysteine up-regulates the expression of p27^kip1^, it does not affect cellular proliferation in Hep3B cells. These results rule out a role of p27^kip1^ for this process.

In the current study, marked induction of ER stress and cell arrest requires supra-physiological concentrations of homocysteine (0.5–1.0 mM) *in vitro*. Previous studies also demonstrate that the concentrations of homocysteine to produce significant effects *in vitro* and *in vivo* are different [41], [18]. The reasonable explanation is that the conditions of *in vitro* studies are different from physiologic conditions [41], [18]. Homocysteine has a short half-life time *in vitro*, whereas the level of homocysteine is constant for a long time *in vivo* [42]. Furthermore, it has been shown that a twofold to sixfold transient increase (approximately 4–12 µmol/g) in intracellular homocysteine, requires an extracellular homocysteine concentration of 1 to 5 mM in HepG2 cells [18]. Thus, Zhang et al. have suggested that it is not the extracellular but the intracellular level of homocysteine that causes ER stress [6].

In conclusion, our study demonstrates that homocysteine inhibits hepatocyte proliferation during hepatic regeneration. Homocysteine up-regulates TRB3 expression through the ER stress pathway, which in turn inhibits the phosphorylation of Akt, resulting in an increase in the protein levels of p53. p53 induces the expression of p21^ Cip1^, thus inhibiting hepatocyte proliferation. These results may enhance our understanding of the direct link between hyperhomocysteinemia and hepatic dysfunction.

## Supporting Information

Figure S1 **Knockdown of p53 inhibits the expression of p2^1cip1^ induced by homocysteine.** (A) HepG2 cells were transfected with control siRNA and siRNA duplexes against p53 mRNA, and incubated with homocysteine (Hcy) (1 mM) for 8 h. The expression of p53 was detected by quantitative RT-PCR. (B) HepG2 cells were transfected with control siRNA and siRNA-p53, and incubated with Hcy (1 mM) for 8 h. The expression of p2^1cip1^ was detected by quantitative RT-PCR.^ a^ *P*<0.05 versus control (without Hcy); ^b^ *P*<0.05 versus Hcy. (C) The proteins were detected by Western blotting. The blot is representative of three independent experiments.
(TIF)

Figure S2 **Knockdown of p53 restores the inhibitory effect of homocysteine on cell proliferation.** HepG2 cells were transfected with control siRNA and siRNA-p53. After incubated with 1 mM of homocysteine (Hcy) for 24 h, the cellular proliferation was assessed by [^3^H]-thymidine incorporation into DNA. These results are means±SD of three experiments.^ a^ *P*<0.05 versus control (without Hcy); ^b^ *P*<0.05 versus Hcy.
(TIF)

Table S1 **The primers for real-time PCR.**
(DOCX)

Methods S1 **RNA interference.**
(DOC)
