# Supplementary figures and images for: Homocysteine Inhibits Hepatocyte Proliferation via Endoplasmic Reticulum Stress
Source: PLoS One. 2013 Jan 22;8(1):e54265. doi: 10.1371/journal.pone.0054265 (PMC3551933; doi:10.1371/journal.pone.0054265)

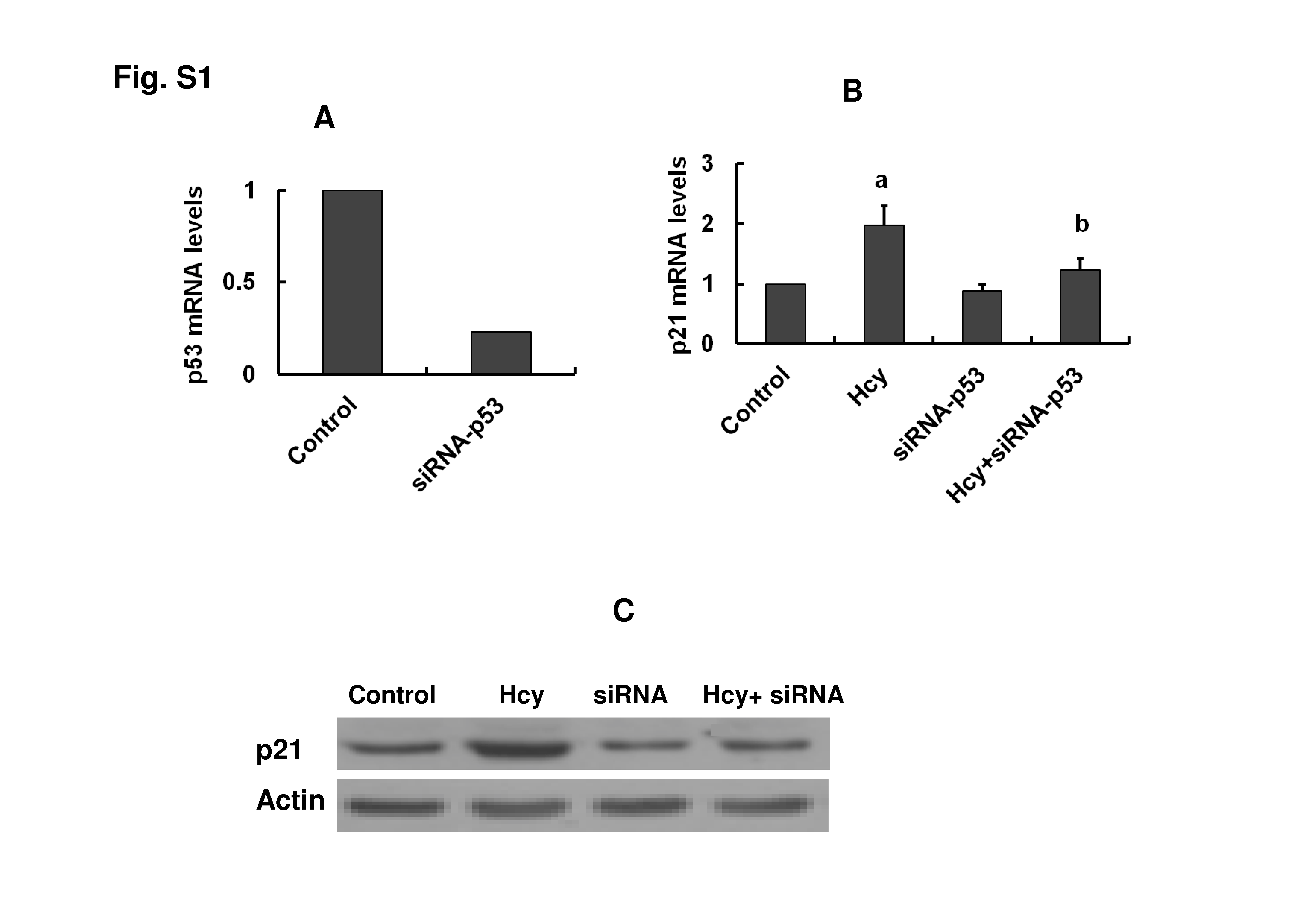

Supplement: Figure S1 — Knockdown of p53 inhibits the expression of p21cip1 induced by homocysteine. (A) HepG2 cells were transfected with control siRNA and siRNA duplexes against p53 mRNA, and incubated with homocysteine (Hcy) (1 mM) for 8 h. The expression of p53 was detected by quantitative RT-PCR. (B) HepG2 cells were transfected with control siRNA and siRNA-p53, and incubated with Hcy (1 mM) for 8 h. The expression of p21cip1 was detected by quantitative RT-PCR. a P<0.05 versus control (without Hcy); b P<0.05 versus Hcy. (C) The proteins were detected by Western blotting. The blot is representative of three independent experiments. (TIF) [file pone.0054265.s001.tif]

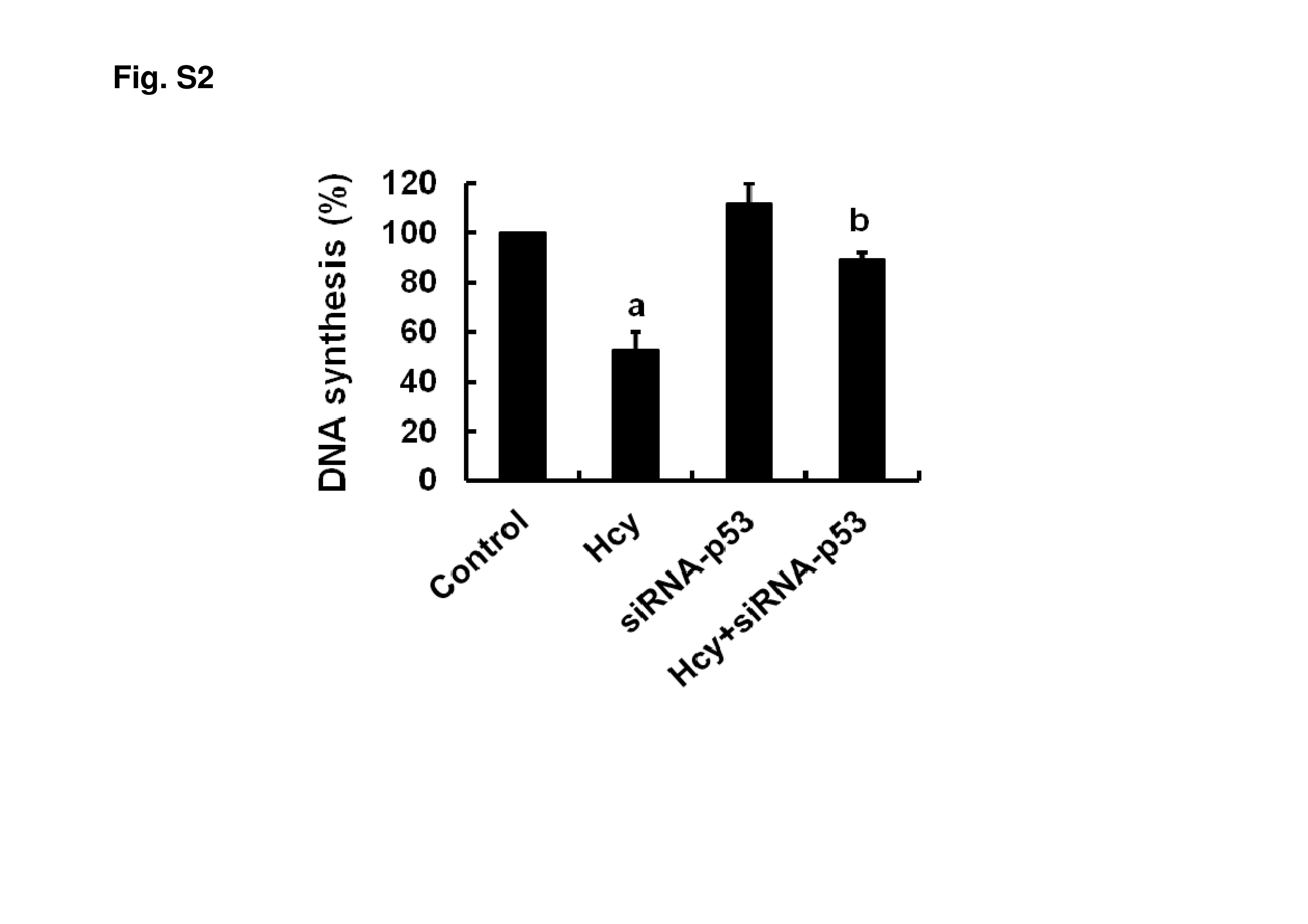

Supplement: Figure S2 — Knockdown of p53 restores the inhibitory effect of homocysteine on cell proliferation. HepG2 cells were transfected with control siRNA and siRNA-p53. After incubated with 1 mM of homocysteine (Hcy) for 24 h, the cellular proliferation was assessed by [3H]-thymidine incorporation into DNA. These results are means±SD of three experiments. a P<0.05 versus control (without Hcy); b P<0.05 versus Hcy. (TIF) [file pone.0054265.s002.tif]
