# Supplementary material for: Homocysteine Inhibits Hepatocyte Proliferation via Endoplasmic Reticulum Stress
Source: PLoS One. 2013 Jan 22;8(1):e54265. doi: 10.1371/journal.pone.0054265 (PMC3551933; doi:10.1371/journal.pone.0054265)
Supplement: Table S1 — The primers for real-time PCR. (DOCX) [file pone.0054265.s003.docx]

**Table S1 The primers for real-time PCR**

| **Human genes** | **Sequences** | **Product length (bp)** |
| --- | --- | --- |
| **CHOP-F**  **CHOP-R** | **AACGGCTCAAGCAGGAAATCG**  **TCTGGGAAAGGTGGGTAGTGTG** | **148** |
| **ATF4-F**  **ATF4-R** | **GACCACGTTGGATGACACTTG**  **GGGAAGAGGTTGTAAGAAGGTG** | **154** |
| **TRB3-F TRB3-R** | **CTGCCCTACAGGCACTGAGTA**  **GAGTGAAAAAGGCGTAGAGGAG** | **155** |
| **p53-F**  **p53-R** | **ACTTGTCGCTCTTGAAGCTAC**  **GATGCGGAGAATCTTTGGAACA** | **113** |
| **p21-F**  **p21-R** | **CCTGTCACTGTCTTGTACCCT**  **GCGTTTGGAGTGGTAGAAATCT** | **130** |
| **p27-F**  **p27-R** | **ATCACAAACCCCTAGAGGGCA**  **GGAGCCCCAATTAAAGGCG** | **169** |
| **GAPDH-F GAPDH-R** | **ACTTTGGTATCGTGGAAGGACTCA**  **GTAGAGGCAGGGATGATGTTCTG** | **133** |

| **Mouse genes** | **Sequences** | **Product length (bp)** |
| --- | --- | --- |
| **CHOP-F**  **CHOP-R** | **TATTGATGCCAAGTGTCCAGTC**  **ACAGCAGGTGACAAGTCTGAG** | **114** |
| **ATF4-F**  **ATF4-R** | **TGCCTTTTCCGGGACAGATTG**  **GTCATCCAACGTGGTCAAGAG** | **124** |
| **TRB3-F TRB3-R** | **GCAAAGCGGCTGATGTCTG**  **AGAGTCGTGGAATGGGTATCTG** | **77** |
| **p21-F**  **p21-R** | **CGAGAACGGTGGAACTTTGAC**  **CAGGGCTCAGGTAGACCTTG** | **106** |
| **p27-F**  **p27-R** | **TCTCTTCGGCCCGGTCAAT**  **GGGGCTTATGATTCTGAAAGTCG** | **116** |
| **p53-F**  **p53-R** | **GTATCCCGAGCATCTGGAAGA**  **CCCCCATGCAGGAGCTATT** | **131** |
| **Bcl-xl-F**  **Bcl-xl-R** | **CTGTGCGTGGAAAGCGTAGA**  **AGGGGCTTGGTTCTTACCCA** | **134** |
| **DR5-F DR5-R** | **GGTGAAGTGGAGCTAAGTCCC**  **CACTGTGCTTTGTACCTGATTCT** | **202** |
| **Actin-F**  **Actin-R** | **AGTGTGACGTTGACATCCGTA**  **GCCAGAGCAGTAATCTCCTTCT** | **112** |
