# Supplementary material for: Homocysteine Inhibits Hepatocyte Proliferation via Endoplasmic Reticulum Stress
Source: PLoS One. 2013 Jan 22;8(1):e54265. doi: 10.1371/journal.pone.0054265 (PMC3551933; doi:10.1371/journal.pone.0054265)
Supplement: Methods S1 — RNA interference. (DOC) [file pone.0054265.s004.doc]

**SI Methods**

**RNA interference**

The siRNA sequence used for depletion of human p53 was that described by Ma and Pederson [1]. p53-targeted siRNAs; sense：5’-AAG ACU CCA GUG GUA AUC UAC dTdT-‘3; antisense：5’-GUA GAU UAC CAC UGG AGU CUU dTdT-3’; and the control siRNAs; sense: 5’-UUC UCC GAA CGU GUC ACG UdTdT-3’; antisense: 5’-ACG UGA CAC GUU CGG AGA AdTdT-3’. siRNAs were purchased from GenePharma Co. (Shanghai, China) and transfected using Lipofectamine 2000 (Invitrogen) according to the manufacturer's protocols.

**References**

1. Ma H, Pederson T (2007) Depletion of the nucleolar protein nucleostemin causes G1 cell cycle arrest via the p53 pathway. Mol Biol Cell 18: 2630–2635
